# Comparative transcriptome analysis provides clues to molecular mechanisms underlying blue-green eggshell color in the Jinding duck (*Anas platyrhynchos*)

**DOI:** 10.1186/s12864-017-4135-2

**Published:** 2017-09-12

**Authors:** Zhepeng Wang, Guohua Meng, Yun Bai, Ruifang Liu, Yu Du, Lihong Su

**Affiliations:** College of Animal Science and Technology, Northwest A&F University, Xinong Road No.22, Yangling, Shaanxi 712100 China

**Keywords:** Duck, Blue-green eggshell color, Biliverdin, Transport activity

## Abstract

**Background:**

In birds, blue-green eggshell color (BGEC) is caused by biliverdin, a bile pigment derived from the degradation of heme and secreted in the eggshell by the shell gland. Functionally, BGEC might promote the paternal investment of males in the nest and eggs. However, little is known about its formation mechanisms. Jinding ducks (*Anas platyrhynchos*) are an ideal breed for research into the mechanisms, in which major birds lay BGEC eggs with minor individuals laying white eggs. Using this breed, this study aimed to provide insight into the mechanisms via comparative transcriptome analysis.

**Results:**

Blue-shelled ducks (BSD) and white-shelled ducks (WSD) were selected from two populations, forming 4 groups (3 ducks/group): BSD1 and WSD1 from population 1 and BSD2 and WSD2 from population 2. Twelve libraries from shell glands were sequenced using the Illumina RNA-seq platform, generating an average of 41 million clean reads per library, of which 55.9% were mapped to the duck reference genome and assembled into 31,542 transcripts. Expression levels of 11,698 genes were successfully compared between all pairs of 4 groups. Of these, 464 candidate genes were differentially expressed between cross-phenotype groups, but not for between same-phenotype groups. Gene Ontology (GO) annotation showed that 390 candidate genes were annotated with 2234 GO terms. No candidate genes were directly involved in biosynthesis or transport of biliverdin. However, the integral components of membrane, metal ion transport, cholesterol biosynthesis, signal transduction, skeletal system development, and chemotaxis were significantly (*P* < 0.05) overrepresented by candidate genes.

**Conclusions:**

This study identified 464 candidate genes associated with duck BGEC, providing valuable information for a better understanding of the mechanisms underlying this trait. Given the involvement of membrane cholesterol contents, ions and ATP levels in modulating the transport activity of bile pigment transporters, the data suggest a potential association between duck BGEC and the transport activity of the related transporters.

**Electronic supplementary material:**

The online version of this article (10.1186/s12864-017-4135-2) contains supplementary material, which is available to authorized users.

## Background

From the blue eggs of the whinchat (*Saxicola rubetra*) to the red eggs of the black-capped donacobius (*Donacobius atricapilla*), from the speckled eggs of the Japanese quail (*Coturnix japonica*) to the eggs of the common murre (*Uria aalge*) with the calligraphic lines, bird eggs display an enormous diversity in eggshell color and maculation pattern, fascinating biological, evolutionary, genetic, and ecological researchers. As a feature exclusively appearing in birds among vertebrates [1], eggshell color can function in camouflage [2], reinforcement of eggshell structure [3], filtering of solar radiation [4], thermal protection [5], or indication of female quality [6], playing important biological roles in ensuring birds successful reproduction. However, the molecular mechanisms underlying these diverse eggshell colors are a poorly understood aspect in avian biology.

Blue-green eggshell color (BGEC) was confirmed to have existed in the extinct upland moa (*Megalapteryx didinus*) [7] and in an oviraptorosaur species [8], suggesting that the color is evolutionarily conserved throughout diverse lineages of Aves [9]. Based on the antioxidant properties of biliverdin, the pigment responsible for BGEC, sexual selection theory indicates that its deposition is related to female antioxidant capacity and egg fertility, inducing males to allocate more parental care in their offspring [6]. There are multiple avian species that lay BGEC eggs, including the whinchat (*Saxicola rubetra*) [9], eastern bluebird (*Sialia sialis*) [10], spotless starling (*Sturnus unicolor*) [11], and some domestic fowls [12–14]. Among these avian species, chickens are unique in that the mutation controlling BGEC was identified as a retrovirus insertion in the *SLCO1B3* gene [15]. Ducks are another type of domestic fowl that lay BGEC eggs. In an earlier study, we have examined expression of *SLCO1B3* in shell glands of blue-shelled ducks and sequence variants. But, we detected neither expression of *SLCO1B3* nor the retrovirus insertion, suggesting that duck BGEC is controlled by different mechanisms [15]. Further identifying the molecular basis of duck BGEC will advance our understanding to the evolution of the trait in the avian phylogeny, and will also improve breeding for the eggshell color in the ducks.

Eggshell color is caused by eggshell pigments. In the final phase of egg formation, the egg enters the shell gland (the uterine part of the oviduct) and stays there for 18–20 h to form eggshell, during which eggshell pigments are secreted into the uterine fluid and progressively deposited in the eggshell, with the deposition rate accelerating in the last 3–5 h before oviposition [16, 17]. Protoporphyrin and biliverdin are two main eggshell pigments and are responsible for red-brown and blue-green eggshell colors, respectively [17]. BGEC is caused predominately by biliverdin-IX and its zinc chelate [17]. Although biliverdin-IX and protoporphyrin simultaneously appear in eggshells of various color, they have different distribution patterns. Protoporphyrin-IX is largely distributed in the cuticle of the eggshell; with heavy scrubbing, the pigment can be removed exposing a white eggshell [18]. In contrast, biliverdin-IX is distributed throughout the eggshell, with the inside of the eggshell being as blue as the outside [12]. This distribution pattern implies that biliverdin deposition should take place together with the calcification of the eggshell.

Biliverdin is a bile pigment that is derived from the oxidative degradation of heme in the reticuloendothelial cells of spleen, liver, bone marrow, and, to a smaller extent, kidney [19, 20]. In mammals, biliverdin is subsequently reduced to bilirubin by biliverdin reductase; the latter is taken up from the blood by hepatocytes, further secreted into bile, and finally excreted in vitro via urine and feces [21]. However, in birds, biliverdin is the predominant end product of heme metabolism due to low biliverdin reductase activity [22]. In terms of the origin of the biliverdin appearing in the eggshell, some researchers believe that it derives from the shell gland, where the pigment is synthesized and secreted [23, 24].

Kennedy and Vevers proposed that BGEC was caused by a specific enzymatic system present in the shell glands of birds laying BGEC eggs that is absent from other birds [24]. At present, it is well known that heme oxygenase (HO) is the rate-limiting enzyme catalyzing the degradation of heme into biliverdin, suggesting that HO is an important candidate for BGEC [19]. In humans and other mammals, HO consists of two active isozymes termed HO-1 and HO-2, which are the products of two different genes, *HMOX1* and *HMOX2*, respectively [19]. HO-1 is an inducible form of HO, and its expression can be induced by all kinds of stimuli and agents that cause oxidative stress and pathological conditions [19]. In contrast, *HMOX2* is constitutively expressed at high levels in the testis and brain [25]. Wang et al. found that *HMOX1* is highly expressed in the shell glands of blue-shelled chickens compared to that in brown-shelled chickens, which suggests that upregulation of *HMOX1* was associated with chicken BGEC [26]. However, it remains unclear whether there is a similar association beween HO expression and duck BGEC.

Apart from this synthesis mechanism, Liu et al. speculated that duck BGEC should be associated with a transport mechanism in the shell gland that allows biliverdin to be more efficiently transported into the uterine fluid, causing BGEC [13]. A similar transport mechanism has been suggested to be present in the chicken [15]. Wang et al. found that chicken BGEC was caused by a retrovirus insertion which activated expression of *SLCO1B3* exclusively in the shell glands of blue-shelled chickens [15]. Functionally, OATP1B3 (gene product) is a membrane transporter that mediates the uptake of bile pigments into the hepatocytes [27]. In birds, biliverdin transport mechanisms are poorly understood to date. In the bile pigment transport system of the liver, it is well known that bilirubin is taken up by hepatocytes via organic anion transporting polypeptide (OATP) and excreted into the bile or blood plasma by multidrug resistance protein (MRP) [28]. OATP1A2, OATP1B3, OATP2B1, MRP1 (*ABCC1*), MRP2 (*ABCC2*), and MRP3 (*ABCC3*) are several known members mediating bilirubin transport [28, 29]. Given their highly similar molecular structures and physicochemical properties [30], biliverdin and bilirubin may share these transporters.

RNA-seq that can sequence the complete set of transcripts in a cell provides an effective method for identifying numerous phenotype-associated differentially expressed genes (DEGs). Using an Illumina RNA-seq platform, a total of 12 libraries (*n* = 3 per group) from the shell glands of two blue-shelled duck (BSD) and two white-shelled duck (WSD) groups were sequenced. Transcriptomic profiles were compared between all pairs of four groups. This study enables identification of BGEC-associated DEGs to establish a sound foundation for analyses into the mechanisms underlying duck BGEC. In addition, massive high quality reads and transcript assemblies reported here provide valuable resources for further annotation of the duck draft genome [31].

## Results

### RNA-seq data and assembly of the transcriptome

**Fig. 1:**
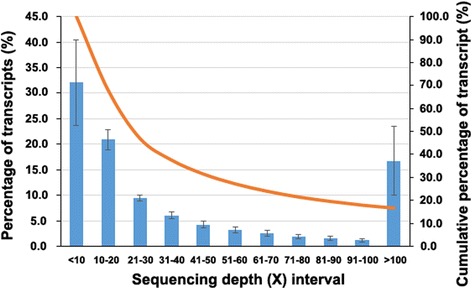
The distribution of the average sequencing depth of transcripts from 12 libraries. Blue bars represent the average percentages of transcripts in a sequencing depth interval. The data was expressed as mean ± SD obtained from 12 libraries. Orange curve represents the cumulative percentages of transcripts from >100 × to <10 ×

A total of 12 libraries (*n* = 3 per group) from 2 BSD and 2 WSD groups were sequenced, generating an average of 40,979,283 ± 10,406,949 clean reads per library (Table 1). An average of 55.9% ± 3.3% of reads were mapped to the duck reference genome (Table 1). The mapping rate was far lower than the ones observed in chicken (79%) [32], mouse (95%) and human (95%) [33]. Given that this RNA-seq generated a great deal of high quality reads as the percentage of Q20 bases showed (Table 1), we speculated that poor assembly quality of currently published duck reference genome should be the main reason for the low mapping rates [34]. Nevertheless, the amount (20.2–36.7 million) of mapped reads remained sufficient to reconstruct full-length transcripts and reliably quantify expression levels for most medium and high abundant genes according to the criteria reported by Martin and Wang [35] and Conesa et al. [36]. These mapped reads were assembled into average 31,542 transcripts (Table 1). However, the transcript number greatly varied, ranging from 22,696 to 39,296 according to samples, because of high variability in the sequencing depth which has a large effect on discovery of novel transcripts and isoforms (Table 1). In addition, biological variability, i.e. samples collected at different stages of shell formation having different transcriptomic profiles [37], can contribute to the variability in transcript number. Of transcripts, 71.3% ± 3.1% were annotated to duck reference genes (Table 1). The N50 of these transcripts was 2849 ± 142 bp. In a de novo assembly study, Zhu et al. reported the N50 of the duck transcriptome assembly to be 329 bp [34]. After the duck reference cDNA was incorporated into the de novo assembly and contigs with lengths shorter than 300 bp were filtered, the N50 was increased to 3214 bp [34]. Therefore, our assemblies based on the duck reference genome were superior to the de novo assembly and approached to the final N50 reported by Zhu et al. [34]*.* The average length of transcripts was 1839 ± 86 bp which was compatible with the average length (1345 bp) of coding sequences reported by Huang et al. [31] (Table 1). The sequencing depths of these assemblies are summarized in Fig. 1. Of these transcripts, 67.9% ± 8.4% were sequenced at a sequencing depth of more than 10 ×, and 16.7% ± 6.4% of transcripts were sequenced at a high depth of more than 100 ×.

**Table 1:** Summary of RNA-seq quality, read counts, mapping rates and transcript assemblies for 12 libraries

Library^a^	Clean read number	Number of clean bases (Gb)	Percentage of Q20 bases (%)	Read mapping rate (%)	Unique hits to reference genome (%)	Number of transcripts	Percentage of annotated transcripts (%)	Mean length (bp) of transcripts	N50 (bp)
BSD1_1	33,901,938	4.28	95.6	59.6	95.7	22,696	71.5	1746	2813
BSD1_2	30,474,832	3.71	96.9	56.2	95.9	25,558	76.1	1784	2703
BSD1_3	31,658,176	3.80	95.8	55.7	96.0	28,311	72.7	1723	2589
WSD1_1	34,289,474	4.33	95.9	59.9	96.5	29,876	74.3	1917	3017
WSD1_2	30,571,674	3.88	96.7	54.5	94.9	31,958	71.6	1972	2923
WSD1_3	36,218,956	4.58	96.1	60.9	96.5	29,373	74.1	1925	2996
BSD2_1	44,488,720	6.67	97.0	54.6	97.2	29,867	72.2	1823	2771
BSD2_2	44,208,500	6.63	97.1	50.8	96.3	28,969	73.3	1936	3025
BSD2_3	66,474,016	9.97	96.9	55.2	97.0	39,296	66.2	1901	3005
WSD2_1	48,196,678	7.23	96.2	52.0	99.2	38,065	67.6	1779	2832
WSD2_2	45,360,764	6.80	95.6	59.1	99.2	37,560	67.7	1755	2752
WSD2_3	45,907,664	6.89	96.1	52.4	99.3	36,978	68.7	1811	2764
Mean ± SD	40,979,283 ± 10,406,949	5.73 ± 1.93	96.3 ± 0.6	55.9 ± 3.3	96.9 ± 1.5	31,542 ± 5308	71.3 ± 3.1	1839 ± 86	2849 ± 142

^a^ *BSD* blue-shelled duck, *WSD* white shelled duck. BSD1_1–3 represent three biological replicates from BSD group 1. Accordingly, BSD2_1–3, WSD1_1–3, and WSD2_1–3 represent three biological replicates from the other three groups

### Identification of BGEC-associated candidate genes

**Fig. 2:**
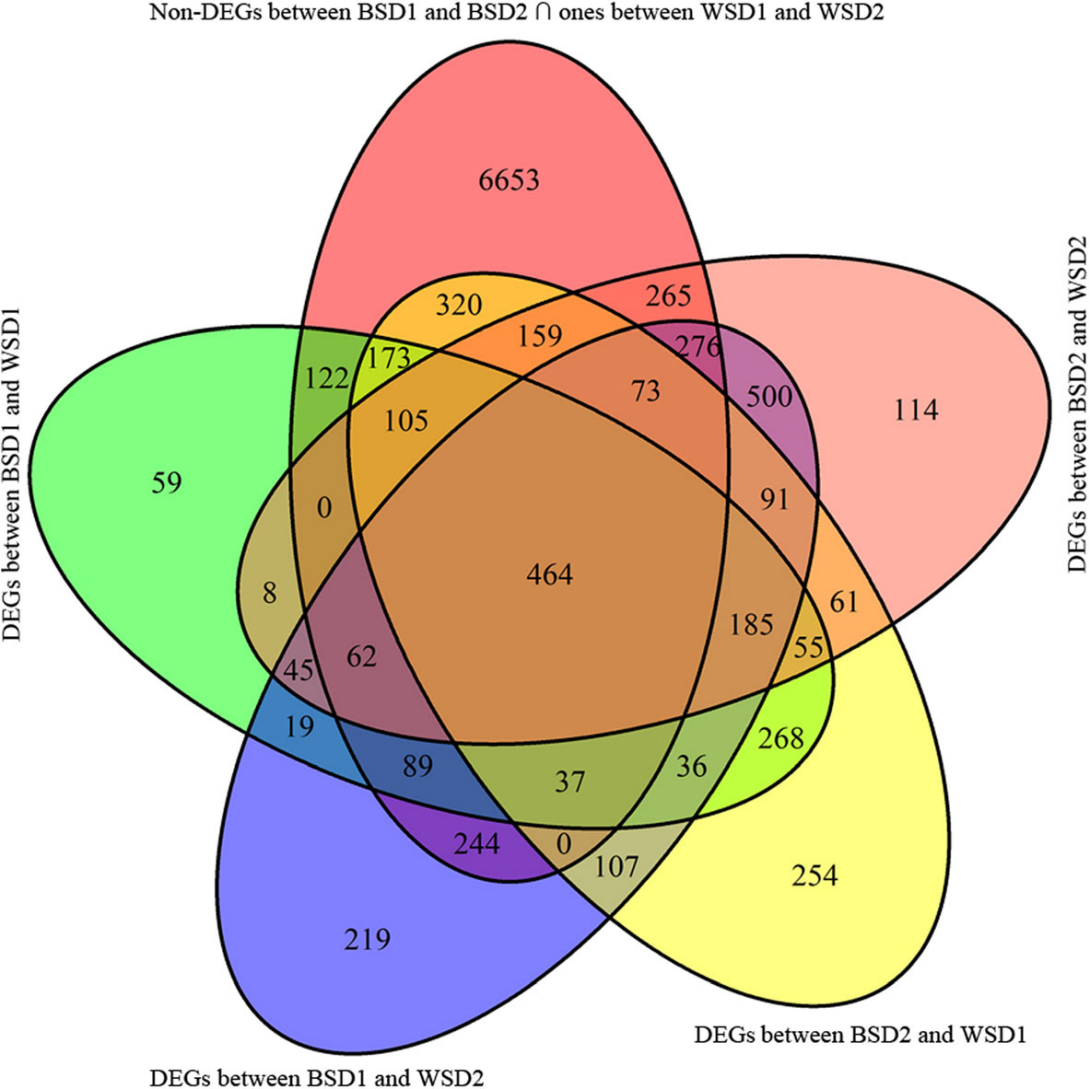
Venn diagram showing the number of shared genes between differentially and non-differentially expressed gene sets. BSD1 and BSD2 represent two blue-shelled duck groups, and accordingly WSD1 and WSD2 indicate two white-shelled duck groups. DEGs represent differenctially expressed genes between BSD and WSD groups; non-DEGs represent non-differentially expressed genes between ducks with the same eggshell color. The numbers in the overlapping area represent the number of shared genes between gene sets, whereas the ones in the single area indicate the number of the genes exclusively appearing in a single gene set. The number of the candidate genes shared between all DEG and non-DEG sets was showed in the center of the venn diagram

To exclude DEGs unrelated to BGEC as much as possible, transcriptomic profiles were compared not only between BSD and WSD groups, but between groups with the same eggshell color. Here, the expression levels of an average 11,698 ± 122 genes were successfully compared between all pairs of 4 duck groups (Additional file 1). Of these genes, 9043 genes consistently showed similar (q > 0.05) expression levels among two same-phenotype comparisons of BSD1 vs BSD2 and WSD1 vs WSD2 (Fig. 2). In contrast, a total of 1726, 2446, 2387, and 2462 genes were differentially (q < 0.05) expressed among the four cross-phenotype comparisons of BSD1 vs. WSD1, BSD1 vs. WSD2, BSD2 vs. WSD1, and BSD2 vs. WSD2 respectively (Fig. 2). A total of 464 genes were shared among the above differentially and non-differentially expressed gene sets, representing promising candidate genes for BGEC (Fig. 2). Of these candidate genes, 230 genes were upregulated in the two BSD groups, and the rest were downregulated. A detailed list of candidate genes is given in Additional file 2.

**Fig. 3:**
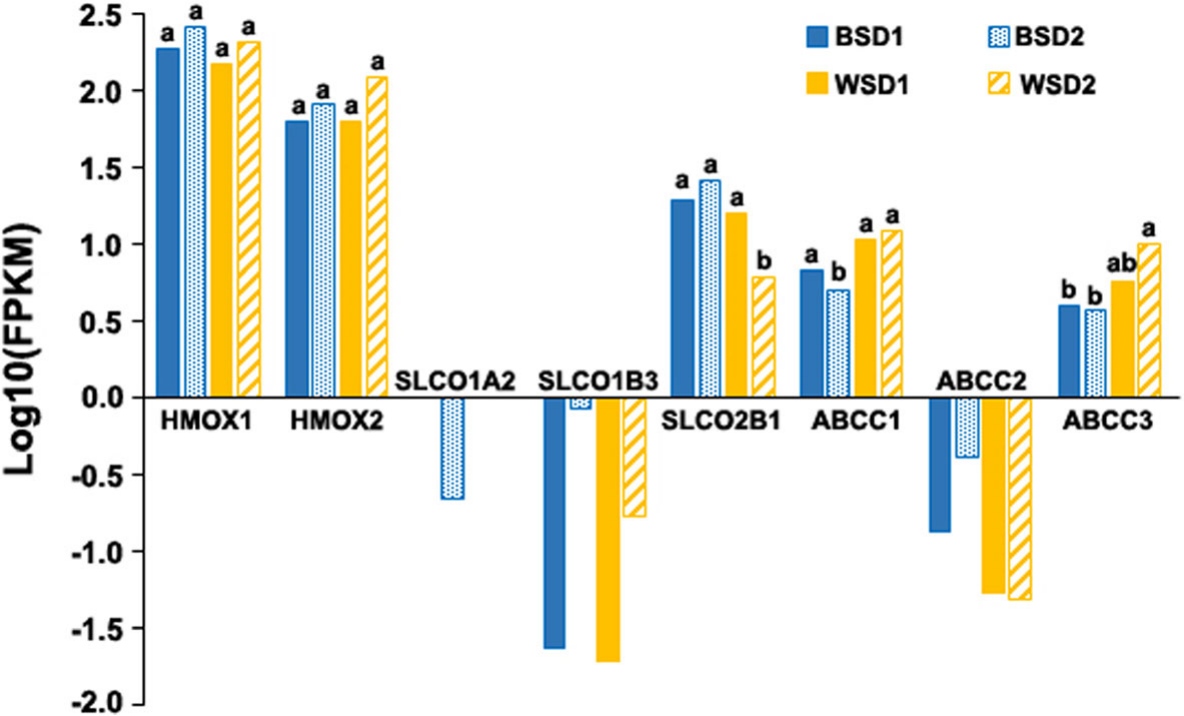
Expression levels of the genes involved in biosynthesis and transport of biliverdin. The histogram shows expression levels of the genes involved in biosynthesis and transport of biliverdin in two blue-shelled (BSD1 and BSD2) and two white-shelled (WSD1 and WSD2) duck groups. Expression levels of the genes in each group were expressed as FPKM values which were cacultated by Cuffdiff using three biological replicates. Letters on bars indicate results of one-side *t*-test between all pairs of four groups; different letters represent significant (q < 0.05) differences between two groups

It is well known that *HMOX1* and *HMOX2* are involved in biliverdin biosynthesis [19] and that some OATP and MRP transporters are responsible for biliverdin transport [28, 29]. However, none of these were present in the list of candidate genes (Additional file 2). Specifically, expression of *SLCO1A2*, *SLCO1B3*, and *ABCC2* was almost negligible (Fig. 3), while expression levels of *HMOX1* and *HMOX2* were not significantly different among four duck groups (Fig. 3). *SLCO2B1*, *ABCC1*, and *ABCC3* did not show a consistent trend in expression among four cross-phenotype comparisons (Fig. 3).

### Functional annotation and overrepresentation analysis of candidate genes

**Fig. 4:**
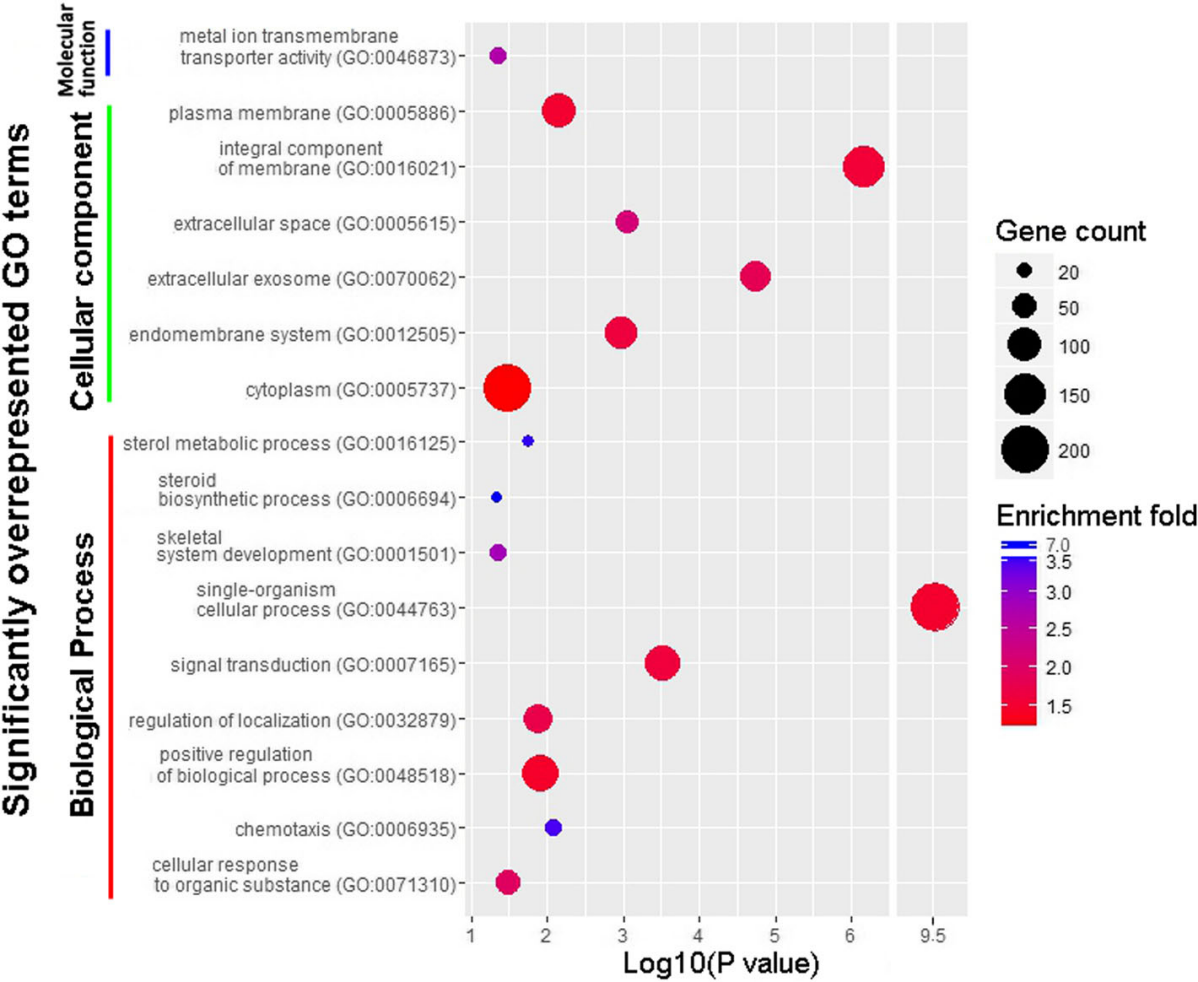
GO-based overrepresentation analysis of candidate genes. The scatter plot shows overrepresentation analysis results of candidate genes. The y-axis shows the significantly (*P* < 0.05) overrepresented GO terms, and x-axis shows the log10 *P* value. The number of candidate genes annotated with a GO term is mapped to the scatter plot by point size. Enrichment folds which were calculated by observed number of candidate genes divided by the expected number are mapped to the graph by different colors; the higher the enrichment fold is, the bluer the color is

After the chicken orthologues of the 464 candidate genes were submitted to the PANTHER Classification System [38], 390 candidate genes were annotated with gene ontology (GO) terms, with 359 candidate genes assigned to 1515 biological process categories, 363 candidate genes annotated with the 250 cellular component categories, and 339 candidate genes classified into 469 molecular function categories (Additional file 3). An overrepresentation test showed that a total of 9 biological process, 6 cellular component, and 1 molecular function categories were significantly (*P* < 0.05) overrepresented (Fig. 4).

**Fig. 5:**
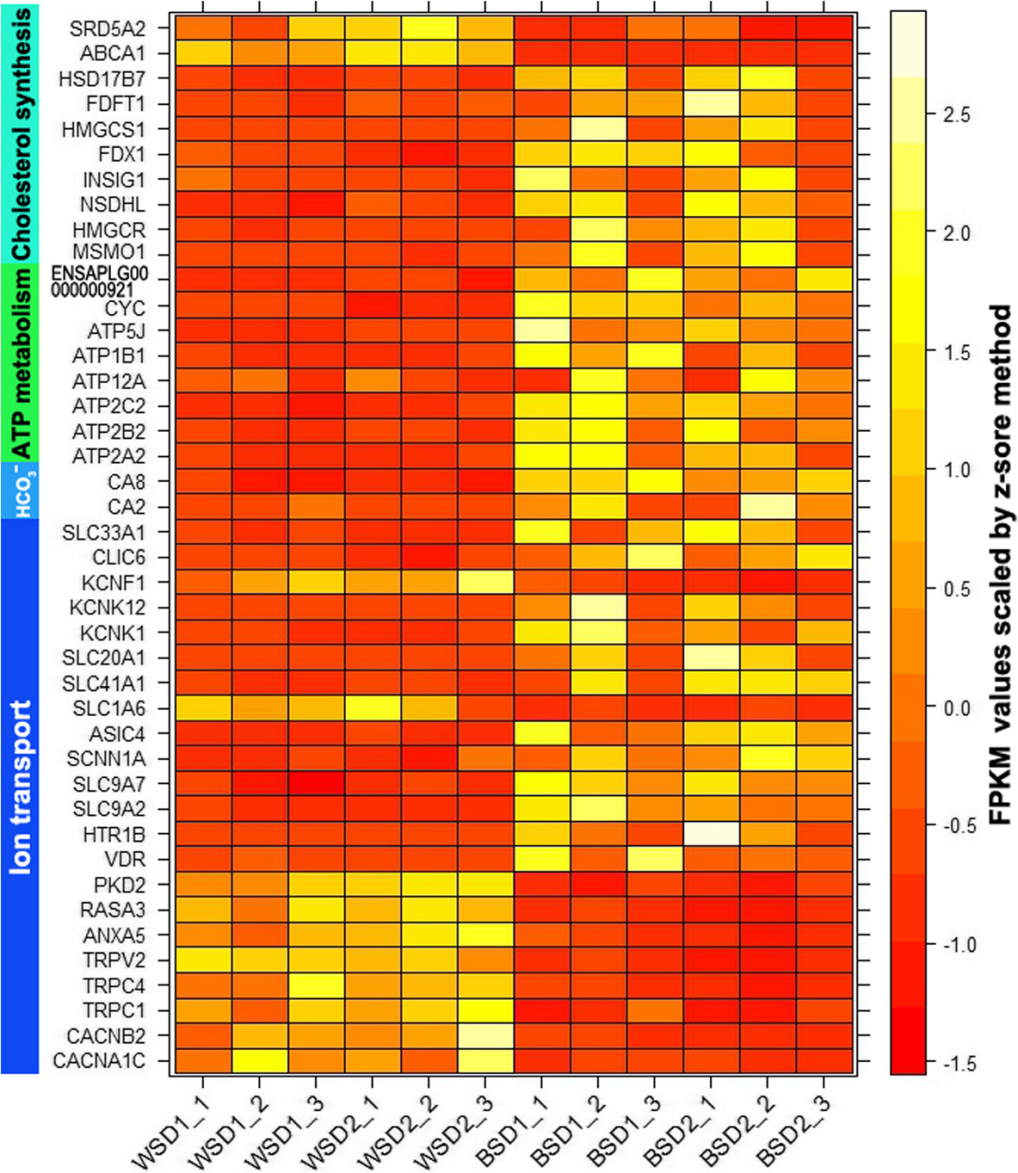
Expression profiles of candidate genes involved in ion transport and cholesterol biosynthesis. The heat map shows expression profiles of candidate genes involved in ion transport, ATP metabolism and cholesterol biosynthesis among 4 duck groups. Each row represents the expression values of one candidate gene; each column represents a biological replicate. Candidate genes were ordered manually according to functional annotations which were shown at the left side of the heat map. More detailed functional annotations were given in Additional file 3. The expression levels of genes were presented as FPKM values. The Z-score scaled FPKM values for each candidate gene were used for plotting. The yellow represents high abundance, and the red means a low expression level. WSD = white-shelled duck, BSD = blue-shelled duck. WSD1_1, WSD1_2, and WSD1_3 represent three biological replicates from WSD group 1. Accordingly, WSD2_1–3, BSD1_1–3, and BSD2_1–3 indicate three biological replicates from the other three groups

Within the biological process categories, the single-organism cellular process (GO:0044763) is a considerably broad GO term with a total of 5353 genes included in this category. Here, the term showed a very low enrichment fold despite over half (221/390) of candidate genes being annotated with the term (Fig. 4). The sterol metabolic process (GO:0016125) and steroid biosynthetic process (GO:0006694) were highly enriched with enrichment folds reaching 6.89 and 7.07, respectively (Fig. 4). There were a total of 10 candidate genes annotated with the two steroid metabolic processes, of which 9 candidate genes were shared by these processes and *ABCA1* was exclusively present in the sterol metabolic process. Differential expression analysis showed that most steroid biosynthetic genes were upregulated in the BSD groups with the exception of *ABCA1* and *SRD5A2* (Fig. 5).

**Fig. 6:**
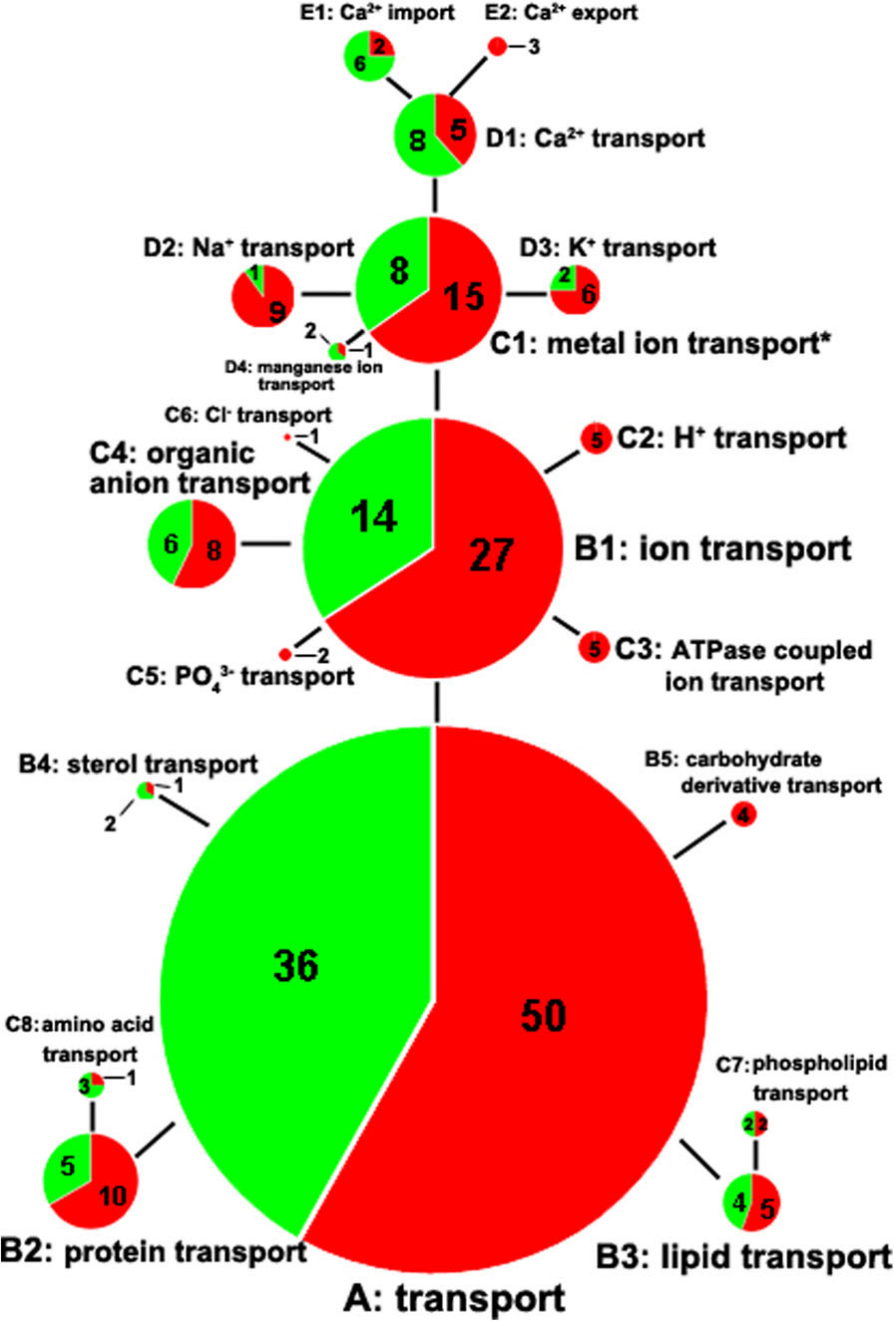
Classification and up- and down-regulated ratios of candidate genes related to transmembrane transport of substances. Up- or down-regulation of candidate genes in the blue-shelled groups was defined relative to the expression levels in the white-shelled groups. The red areas represent the ratios of upregulated genes, and the green for the downregulated ratios. The numbers of up- and downregulated genes are showed in the pie graphs. Letters from A to E near the pie graphs represent different hierarchies of transport-related functions; Letter “A” represents the most general classification, and “E” represents the most specific classification. Brief descriptions for each classification were given in the figure. Detailed GO annotations and lists of candidate genes were summarized in Additional file 5. Asterisk (*) in the upper right of C1 indicated that the GO category was significantly (*P* < 0.05) overrepresented

Among the cellular component categories, the integral component of membrane (GO:0016021) represented a highly significant (*P* = 6.81e-7) and large category, with 153 candidate genes included (Fig. 4). However, a low enrichment fold was observed for the term like the situation in the single-organism cellular process (Fig. 4). Integral components of the membrane, in other words, membrane proteins, exhibit multiple biological roles in transport, signal transduction, cell adhesion and recognition, and energy transduction [39]. Given that the main function of the shell gland is to form the eggshell, which requires the transmembrane transport of multiple ions, matrix proteins, and eggshell pigments, the transport functions of membrane proteins are of particular interest. Of 153 candidate genes, 86 candidate genes were annotated with the transport term (GO:0006810) and are expected to mediate the transmembrane transport of proteins, amino acids, lipids, carbohydrate derivatives, sterols and various types of ions (Fig. 6). Ion transport (GO:0006811) represented the largest transport category, into which almost half (41/86) of the transport-related candidate genes were assigned (Fig. 6). Furthermore, the metal ion transport was significantly overrepresented by 23 candidate genes (Fig. 4). Differential expression analysis showed that most transport genes were upregulated in the BSD groups with the exception of calcium ion, manganese ion, sterol, amino acid transport genes (Fig. 6).

### Expression analysis of ion transporters related to eggshell mineralization

During eggshell formation, a series of ions, including Ca^2+^, HCO_3_^−^, Na^+^, K^+^, H^+^, and Cl^−^ participate in the mineralization process [40]. Here, a total of 13 Ca^2+^, 10 Na^+^, 8 K^+^, 5 H^+^, and 1 Cl^−^ transporter and two HCO_3_^−^ synthesis genes were identified among the candidate genes (Additional files 2, and 4). Among the 13 Ca^2+^ transport genes, *ATP2C2*, *HTP1B*, *PKD2*, *RASA3*, *CACNB2*, *TRPC1*, *TRPC4*, and *TRPV2* were further annotated as calcium ion import (GO:0070509) and positive regulation of calcium ion import (GO:0090280) (Additional file 5). These Ca^2+^ import genes were almost all downregulated in the BSD groups, with the exception of *HTR1B* and *ATP2C2* (Fig. 5). In contrast, three Ca^2+^ pumps (*ATP2A2*, *ATP2B2*, and *ATP2C2*) were all upregulated (Fig. 5). Genes mediating Na^+^, K^+^, H^+^, and Cl^−^ transport were almost all upregulated, with the exception of one Na^+^ (PKD2) and two K^+^ (PKD2 and KCNF1) transport genes (Fig. 5). In addition, two HCO_3_^−^ synthesis genes (*CA2 and CA8*) were upregulated in the BSD groups (Fig. 5).

We identified a considerable number of candidate genes involved in ion transport coupled ATP hydrolysis (i.e. Ca^2+^ pumps and Na^+^/K^+^ exchangers) and synthesis (i.e. H^+^ transporters). We found five ion transporters (*ATP12*, *ATP1B1*, *ATP2A2*, *ATP2B2*, and *ATP2C2*) with ATPase coupled ion transmembrane transporter activity (GO:0042625), one H^+^ transporter (ATP5J) with ATP synthesis coupled proton transport (GO:0015986), and two candidate genes (ENSAPLG00000000921 and CYC) with mitochondrial ATP synthesis coupled electron transport (GO:0042775). The above ATP metabolism genes were all upregulated in the BSD groups (Fig. 5).

### Verification of DEGs by qPCR

RNA-seq data were verified by qPCR. A total of nine genes were selected for verification, representing three expression types: (1) *SLCO1A2*, *SLCO1B3*, and *ABCC2*, which were only barely detected by RNA-seq (Fig. 3); (2) *HMOX1* and *ABCC1*, which were expressed, but did not show a significant expression difference or a consistent trend among four cross-phenotype comparisons (Fig. 3); (3) *CA2*, *ATP1B1*, *ATP2B2* and *SLC14A1*, four candidate genes that were all upregulated in the BSD groups (Additional file 2). Expression of *SLCO1A2*, *SLCO1B3*, and *ABCC2* remained undetected by qPCR (Additional file 6). Expression of the other seven genes was consistent with the RNA-seq results (Additional file 6). The correlation coefficient between qPCR and RNA-seq was 0.914 (*P* < 0.01), indicating good reproducibility of the differential expression results obtained by RNA-seq (Additional file 6).

## Discussion

Eggshell color, a principle component of avian reproductive system, is related to reproduction in wild birds [1], and to customer preference [41, 42] and egg nutrition [43, 44] in domestic fowls. Although considerable research efforts have focused on the biological roles of eggshell color and the biochemistry of eggshell pigments [17, 24, 45], molecular mechanisms underlying eggshell colors are poorly understood so far. Using the Illumina RNA-seq, we obtained a global view of transcriptomes from shell glands of BSD and WSD. Further, expression levels of 11,698 genes were successfully compared between all pairs of two BSD and two WSD groups, resulting in the discoveries of 464 BGEC-associated candidate genes. This study is among the first to employ RNA-seq to robustly discover BGEC-associated DEGs, further to provide insight into the molecular mechanisms.

BGEC is caused by biliverdin [17]. Thus, any mechanisms that enhance the biosynthesis or transport efficiency of the pigment in the shell gland can cause BGEC. Here, the expression of two biliverdin biosynthesis genes, *HMOX1* and *HMOX2*, was not significantly different between BSD and WSD groups. This result is compatible with the finding of Liu et al. that BSD and WSD have similar enzymatic activities of HO in the shell glands [13], suggesting that duck BGEC is not caused by a biliverdin synthesis mechanism.

In all kingdoms of life, anomalous transport of pigments themselves or pigment-related substrates is another important pigmentation mechanism, which has been confirmed to be responsible for eye color in *D.melanogaster* [46] and silkworm [47]; shell color in pacific oyster [48]; and coat color in horse [49], tiger [50], and chicken [51]. A pigment transport mechanism responsible for bird BGEC has been proposed by Liu et al. [13] and Wang et al. [15]. OATP1A2, OATP1B3, OATP2B1, MRP1, MRP2 and MRP3 are well-known transporters mediating bile pigment transport [28, 29]. It is therefore tempting to speculate that upregulation of these transporters increase transport efficiency of biliverdin, further causing duck BGEC. But, current results did not obviously support the speculation (Fig. 3). However, there is another possibility that it is due to transport activity rather than to upregulation of transporters that duck BGEC is caused.

Cholesterol contents in the membrane have a great effect on the activities of bile salt transporters. A well-characterized example is derived from the association of intrahepatic cholestasis with the mutations in *ATP8B1* [52]. *ATP8B1* is a member of P4 ATPase family and exerts an important role in resisting bile salt-mediated cholesterol extraction from the canalicular membrane [52]. ATP8B1 deficiency reduces cholesterol contents in the canalicular membrane, which impairs the activities of the bile salt export pumps, further causing the cholestasis [52]. In this study, we found that the steroid biosynthsis process was highly enriched by 9 candidate genes which were almost all upregulated in the BSD except for SRD5A2 (Fig. 5). ABCA1 that mediates cholesterol export in peripheral tissues was downregulated in the BSD (Fig. 5) [53]. Although the exact interaction mechanisms of cholesterol and biliverdin transporters have not been fully understood, our results and the known relationship between membrane cholesterol contents and the transport activity of bile salt transporters urge us to speculate that upregulation of cholesterol biosynthesis genes and downregulation of a cholesterol export gene (ABCA1) may exert a positive action for membrane cholesterol contents, which promotes the activity of biliverdin transporters logically.

Apart from the potential cholesterol-related mechanism, ion transport can be implicated into the formation of BGEC. Eggshell mineralization refers to transmembrane transfers of multiple ions including Ca^2+^, HCO_3_^−^, Na^+^, K^+^, H^+^, and Cl^−^ [40]. During the process, concentrations of Ca^2+^, K^+^ and H^+^ in the uterine fluid continuously increase from 8 h to 18 h post ovulation; ones of Na^+^ and Cl^−^ generally decline [54]. A similar increasing process has been observed for biliverdin from 12 h to 23.5 h post ovulation [13]. The synchronism implies that an ion/biliverdin exchange or symport mechanism can exist in the shell glands of the birds laying BGEC eggs, by which biliverdin deposition can occur together with eggshell mineralization.

**Fig. 7:**
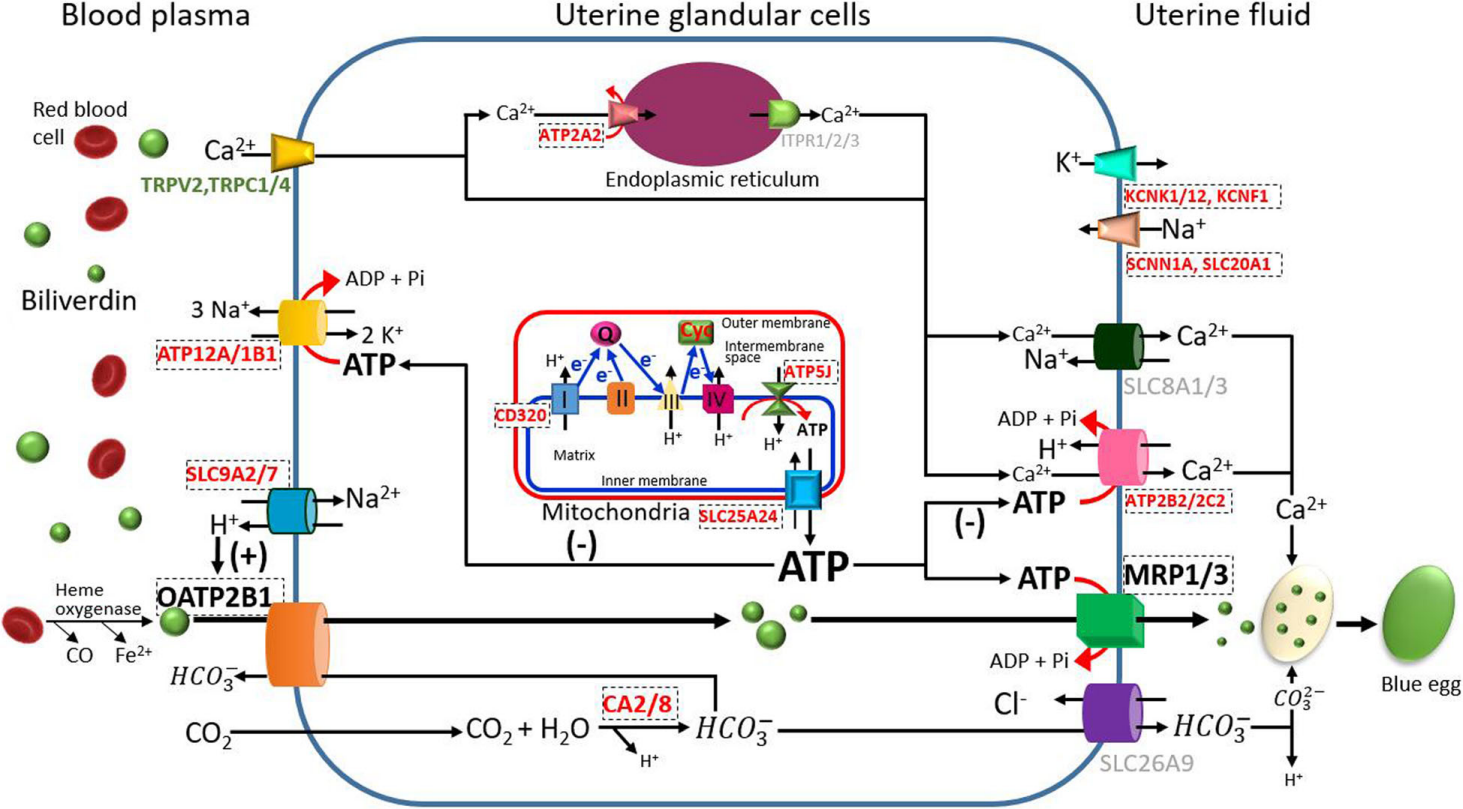
A balance hypothesis explaining that differential expression of ion transporters can cause blue-green eggshell color. Given the uncertainties in the cellular localizations, transfer directions, and ion types for some ion transporters, a large part but not all of differentially expressed ion transporters were incorporated in the balance model. Cellular localizations of ion transporters were determined according to the ion transfer model proposed by Jonchère et al. [40] and GO annotations. Transporter names shown in red represent that they were upregulated in the blue-shelled groups; the green indicate downregulation; the gray mean that the transporters were incorporated in the Jonchère’s model [40] but were not detected by this RNA-seq; ones shown in bold black letters are several potential biliverdin transporters which were expressed in shell glands of both blue-shelled and white-shelled ducks but did not show consistent expression trends among all cross-phenotype comparisons. The sign of “(+)” represents that upregulation of two H^+^ extruders can stimulate the transport activity of OATP2B1; the sign of “(−)” indicates that a competition for ATP can exist between multidrug resistance protein 1/3 (MRP1/3) and ion pumps (ATP2A2, ATP2B2, ATP2C2, ATP1B1, and ATP12A). The letters of “Q”, “I”, “II”, “III”, “IV”, and “Cyc” respectively represent coenzyme Q, complex I-IV, and cytochrome c which are involved in the mitochondria electron transport. CD320 is the chicken orthologue of duck ENSAPLG00000000921. Because the gene was annotated with the mitochondrial electron transport, NADH to ubiquinone (GO:0006120), it can exert a role in the first step of the electron transport chain

It is believed that OATP-mediating uptake of bilirubin is completed by an anion-exchange mechanism, and HCO_3_^−^ is the first identified counter ion [27]. In addition, the transport activity of some OATPs, i.e. OATP1C1, OATP2B1, is stimulated by an extracellular acidic milieu, and adjacent acid extruders, i.e. Na^+^/H^+^ exchangers, are potential modulators [27]. MRP-mediating bilirubin efflux is an active transport process dependent on ATP [28]. Here, we found two Na^+^/H^+^ exchanger (*SLC9A2* and *SLC9A7*), two HCO_3_^−^ synthesis (*CA2* and *CA8*) and eight ATP metabolic (*ATP1B1*, *ATP2A2*, *ATP2B2*, *ATP2C2*, *ATP12A*, *ATP5J*, *CD320* and *CYC*) genes in the list of candidate genes. Given the close relationship between biliverdin transport and HCO_3_^−^, pH, and ATP, these candidate genes may cause BGEC by regulating the transport activity. We propose a balance hypothesis to explain the possibility (Fig. 7).

Firstly, ion transporters have been suggested to be the promising candidates for the eggshell mechanical properties [55]. In this study, although a considerable number of ion transporters were differentially expressed between BSD and WSD, there is no evidence supporting that blue and white duck eggs have a significant difference in eggshell quality. The absence of difference between blue and white eggs has been confirmed in the chicken [56].

Secondly, CA2 and CA8 were upregulated in the BSD (Fig. 5), which can increase the yield of intracellular HCO_3_^−^. Redundant HCO_3_^−^ can be transported into the blood plasma via OATP2B1 (Fig. 7). The consequence of balancing the concentration of intracellular HCO_3_^−^ is that more biliverdin is exchanged into glandular cells (Fig. 7). In addition, given that two Na^+^/H^+^ exchangers were upregulated, an acidic extracellular pH may further enhance the transport activity of OATP2B1 (Fig. 7).

Thirdly, there were three Ca^2+^ (*ATP2A2*/*2B2*/*2C2*) and two Na^+^ (*ATP1B1*, *ATP12A*) pump, three ATP synthetic (*ATP5J*, ENSAPLG00000000921 and *CYC*) and one ATP transport (*SLC25A24*) genes which were upregulated in the BSD among 464 candidate genes (Fig. 5). To maintain intracellular ion and ATP homeostasis, potential effect caused by upregulation of these genes can be balanced by a competition for ATP between ion pumps and MRPs. The consequence of the competition may be more biliverdin transferred into the uterine fluid, finally causing the BGEC (Fig. 7).

As we know, administration of coccidiostat Nicarbazin leads to eggshell depigmentation [17], and acetazolamide suppresses the activity of carbonic anhydrases [57]. Therefore, it will be helpful to accurately characterize the association of these candidate genes with duck BGEC, and to establish their relative contribution by incorporating the inhibitory models in the future study.

## Conclusions

This study found 464 candidate genes potentially associated with duck BGEC whereas none of them were directly implicated into biliverdin biosynthesis or transport. In contrast, cholesterol biosynthsis and ion transport processes were significantly overrepresented by candidate genes. Given potential roles of membrane cholesterol contents, ions and intracellular ATP levels in modulating the transport activity of bile pigment transporters, the data suggests that duck BGEC might be associated with the change of the transport activity of the related transporters. Our present and earlier [15] results support the possibility that the abnormal transport of biliverdin may be shared by avian species laying BGEC eggs despite of the causative mutations probably differing from each other. Sexual selection theory interpreted BGEC as a signal reflecting the genetic quality of the females [6]. Our balance hypothesis speculates that BGEC is a consequence of balancing potential effect caused by differential expression of ion transport and synthesis genes, supporting that a structural significance can exist for BGEC.

Even though the currently available duck genome annotation has been shown to be quite incomplete [34], we are thus sure to have implemented the best approach we could. A new, much improved duck genome reference with annotation is underway by international efforts but will only become available in the future. Our data of 492 million high quality reads and assembled transcripts will aid significantly in the annotation efforts by enriching the databases during future annotations.

## Methods

### Ducks and collection of shell glands

A Chinese indigenous breed, Jinding duck (*Anas platyrhynchos*), was used in this study. Jinding duck is originated from the Fujiang province of China, and characterized by an excellent laying performance [58]. In the Jinding ducks, the eggshell color is not fixed, where major individuals lay BGEC eggs, but there are minor ducks laying white eggs. Two Jinding duck populations were independently raised in two duck farms at the Baiyang Lake of HeBei province of China. There was no gene flow between the two duck populations. Three blue-shelled and three white-shelled ducks were randomly selected from population 1, and the other three blue-shelled and three white-shelled ducks were collected from population 2. In the text, blue- and white-shelled ducks from population 1 were respectively defined as BSD1 and WSD1, and ducks from population 2 were named as BSD2 and WSD2. All birds were 35 weeks old and had access to food and water ad libitum.

Before sampling, a part of the ducks were caged individually. The oviposition was monitored every 30 min. According to the oviposition routines, ducks were stunned and killed at 3–5 h before estimated oviposition. Shell glands (the uterine part of the oviduct) were excised, rinsed briefly with 0.9% isotonic saline and immediately immersed in RNAstore RNA stabilization reagent (CWBIO) overnight at 4 °C and finally stored at −80 °C. The care and use of ducks and collection of shell glands were performed according to the guidelines of the Animal Care and Use Committee of Northwest A&F University.

### RNA-Seq library preparation and Illumina-Solexa sequencing

Total RNA was extracted from shell glands with TRIzol reagent (Invitrogen) according to the manufacture’s protocol. The sequencing libraries were prepared for each duck using NEBNext® Ultra™ RNA Library Prep Kit (NEB). Briefly, mRNA was isolated from total RNA with poly-T oligo-attached magnetic beads and then fragmented. First-strand cDNA was synthesized using M-MuLV reverse transcriptase (RNase H-) and random primers. The cDNA was further converted into double stranded cDNA. Following the reverse transcription, the cDNA was end-repaired, adenylated, and ligated to NEBNext Adaptors. The cDNA fragments of 150–200 bp in length were selected with AMPure XP system (Beckman Coulter). The size-selected, adaptor-ligated cDNA was treated with 3 μL USER enzyme (NEB). PCR was performed with Phusion High-Fidelity DNA polymerase (NEB), universal PCR primers and Index (X) Primer. Clustering of the index-coded samples was performed on a cBot Cluster Generation System using TruSeq PE Cluster Kit v3-cBot-HS (Illumia) according to the manufacturer’s instructions. After the clustering was completed, these libraries were sequenced using a paired-end 2 × 125 bp lane (BSD1 and WSD1) and a 2 × 150 bp lane (BSD2 and WSD2) on an Illumina HiSeq 2500 platform.

### Acquisition and analysis of RNA-Seq data

A total of 12 libraries (*n* = 3 per group) from shell glands of two BSD and two WSD groups were sequenced. Raw reads of fastq format were firstly filtered to generate clean reads by removing reads containing adapters or ambiguous nucleotides (the proportion of “N” base exceeding 10%), and reads of low quality (the proportion of bases with Phred quality score of less than 20 exceeding 50%). The filtering steps were completed by Novogene Co. (Beijing, China) using their in-house Perl scripts. The duck reference genome (BGI duck 1.0 Assembly, release-82) and gene model annotation files (GTF file) were downloaded from Ensembl database [59, 60]. Indexes of the reference genome were built using Bowtie v2.2.7 [61]. The paired-end clean reads were aligned to the reference genome by Tophat v2.0.14 using the parameters described by Tropnell C et al. with little modification made in the parameter of N that was set to four [62].

Following alignment, mapped reads were handled by Cufflinks v2.2.0 to generate a transcriptome assembly for each library. All assemblies from 12 libraries and reference transcripts were merged by Cuffmerge to generate a uniform basis for calculating transcript and gene expression [62]. Finally, the mapped reads, the merged assembly, and the duck reference genome were submitted to Cuffdiff to calculate expression levels, run bias detection and correction algorithm, and test the statistical significance of changes in abundance of each gene between all pairs of 4 groups [62].

Cuffdiff estimates abundance at the transcript level using a linear statistical model [62]. The abundance of each gene is calculated by adding up the expression levels of a group of transcripts from the gene [62]. Abundance is reported as fragments per kilobase of transcript per million mapped fragments (FPKM), which is calculated according to the number of reads uniquely mapped to the gene [62]. In this method, read counts are normalized by gene length, and then by sequencing depth, which ensures that abundance estimates are proportional to the number of reads produced by each gene and are less influenced by these variables [62, 63].

When more than two groups are handled, Cuffdiff performs all possible pair-wise comparisons between these groups [64]. To test for significant differences, the square root of the Jensen-Shannon divergence of the relative abundances in each of two groups is calculated. The variance of this Jensen-Shannon distance under the null hypothesis of no change in relative abundance is estimated. Using the estimated variance, one-sided *t*-test is performed to evaluate the significance in the abundance changes of each gene between two groups [65]. The *P*-value was adjusted using the Benjamini and Hochberg’s approach for controlling type I errors due to multiple testing [65]. Genes with a q value <0.05 were determined to be differentially expressed.

### Screening of candidate genes and functional annotation

Candidate genes were obtained according to three conditions as follows: (1) an assembled gene was annotated to unique one reference gene; (2) gene expression was detected among the four duck groups, and differential expression analysis was successfully performed across all six pair-wise comparisons; (3) expression levels of genes were consistently significantly different between all cross-phenotype groups (BSD1 vs. WSD1, BSD1 vs. WSD2, BSD2 vs. WSD1 and BSD2 vs. WSD2), but not for between same-phenotype groups (BSD1 vs. BSD2 and WSD1 vs. WSD2).

Functional annotation and overrepresentation tests of candidate genes were completed using the PANTHER Classification System [38]. Complete GO molecular function, biological process, and cellular component categories were used as function annotation and overrepresentation analysis data sets. Because the duck genes have not been deposited in the PANTHER Classification System to date, chicken orthologues of these candidate genes (Additional file 7) were submitted to the PANTHER for functional analysis. Chicken orthologues were found by BLAT alignment between duck genes and chicken genome (Galgal4, Ensembl release 82), with a cut-off E-value of 0.00001 [66]. The genes with the highest alignment score were identified as the chicken orthologues. Overrepresentation tests against the chicken genome background were performed using the statistical overrepresentation test option. Statistical significance was assessed using the binomial test. Bonferroni correction was performed to control type I errors caused by multiple tests. GO terms with an adjusted *P* value less than 0.05 were considered to be significantly overrepresented. If there was an ancestor-child hierarchy relationship among significant GO terms, only the most specific child terms were considered.

### Real-time quantitative RT-PCR (qPCR) verification of RNA-seq data

The RNA-seq data were verified by qPCR using the same RNA samples used for RNA-seq. The concentration of total RNA was measured using a NanoDrop 2000c spectrophotometer (Thermo Fisher Scientific). One μg of RNA was used as a template from which first-strand cDNA was synthesized using a SuperRT cDNA Synthesis Kit (CWBIO). QPCR was performed using an UltraSYBR Mixture Kit (CWBIO). The total volume of the qPCR mixture was 20 μL containing 10 μL of 2 × UltraSYBR Mixture (High ROX), 0.4 μL of forward primer (10 μM), 0.4 μL of reverse primer (10 μM), 1 μL of cDNA, and 8.2 μL of ddH_2_O. Reactions were carried out on a Bio-Rad iQ™5 Thermal Cycler (Bio-Rad) with the following reaction conditions: denaturation at 95 °C for 10 min; 40 cycles of denaturation at 95 °C for 15 s, annealing and elongation for 1 min at 60 °C, and a melting curve process from 55 to 95 °C. Nine genes were selected as qPCR-verified targets. The primer sequences for these genes were listed in Additional file 8. Three biological replicates per groups and two technological replicates per sample were included in the qPCR. *GAPDH* was used as the endogenous reference gene, and the WSD2 group was set as the criterion. Expression levels of genes in each group were presented as expression folds relative to the criterion using the 2^-ΔΔCT^ method [67].

## Additional files

Additional file 1: A whole list of genes, of which expression has been successfully compared between all pairs of 4 duck groups. BSD1 & 2 represent blue-shelled duck group 1 & 2, and WSD1 & 2 indicate white-shelled duck group 1 & 2. Expression levels of genes in each group were presented as FPKM values which were calculated by Cuffdiff using three biological replicates per group. Changes of abundance between all pairs of 4 groups were tested using one-side *t*-test. A q value of less than 0.05 indicated that a change of abundance was significantly different between two groups. (XLSX 6970 kb)

Additional file 2: Differential expression analysis results of 464 candidate genes between all pairs of 4 duck groups. These candidate genes were consistently differentially (q < 0.05) expressed between all cross-phenotype groups (BSD1 vs. WSD1, BSD1 vs. WSD2, BSD2 vs. WSD1, and BSD2 vs. WSD2), but not for between same-phenotype groups (WSD1 vs. WSD2 and BSD1 vs. BSD2). A q value of less than 0.05 indicated that a change of abundance was significantly different between two groups. The upregulated genes in the BSD groups were highlighted in red background and the downregulated ones were indicated in green background. BSD1 & 2 = blue-shelled duck group 1 & 2, WSD1 & 2 = white-shelled duck group 1 & 2. (XLSX 282 kb)

Additional file 3: Complete gene ontology (GO) annotations for candidate genes. The chicken orthologues of these candidate genes were submitted to the PANTHER to complete the functional annotation [38]. There were a total of 390 candidate genes annotated successfully with GO terms among 464 candidate genes. The rest 74 genes either were not found in the PANTHER or were not annotated with any GO terms. (XLSX 109 kb)

Additional file 4: Table S1. Information summary for ion transport and synthesis genes potentially associated with eggshell mineralization. (XLSX 17 kb)

Additional file 5: A detailed list of candidate genes related to the transport of various substances. GO annotations of candidate genes were completed using the PANTHER classification system [38]. White-shelled duck groups were designed as the criterion. Upregulation or downregulation of candidate genes in the blue-shelled duck groups was given relative to the criterion. (XLSX 51 kb)

Additional file 6: Figure S1. Verification of RNA-seq results using qPCR. BSD1 & BSD2 respectively indicate two blue-shelled duck groups; accordingly WSD1 & WSD2 represent two white-shelled duck groups. In the RNA-seq, FPKM values in each group were calculated by Cuffdiff using three biological replicates. In the qPCR, the WDS2 group was set as the criterion; the abundances of genes in each group were presented as expression folds relative to the criterion. The qPCR results were expressed as mean ± SD obtained from 3 biological replicates. The correlation plot showed the relationship between qPCR and RNA-seq results of *HMOX1*, *ABCC1*, *CA2*, *ATP1B1*, *ATP2B2* and *SLC14A1*. ** indicates a significant correlation relationship at *P* < 0.01. (JPEG 367 kb)

Additional file 7: 464 duck candidate genes and corresponding chicken orthologues. (XLSX 28 kb)

Additional file 8 Primer sequences used in the real-time quantitative RT-PCR verification. (XLSX 15 kb)
