# Supplementary figures and images for: Comparative transcriptome analysis provides clues to molecular mechanisms underlying blue-green eggshell color in the Jinding duck (Anas platyrhynchos)
Source: BMC Genomics. 2017 Sep 12;18:725. doi: 10.1186/s12864-017-4135-2 (PMC5596863; doi:10.1186/s12864-017-4135-2)

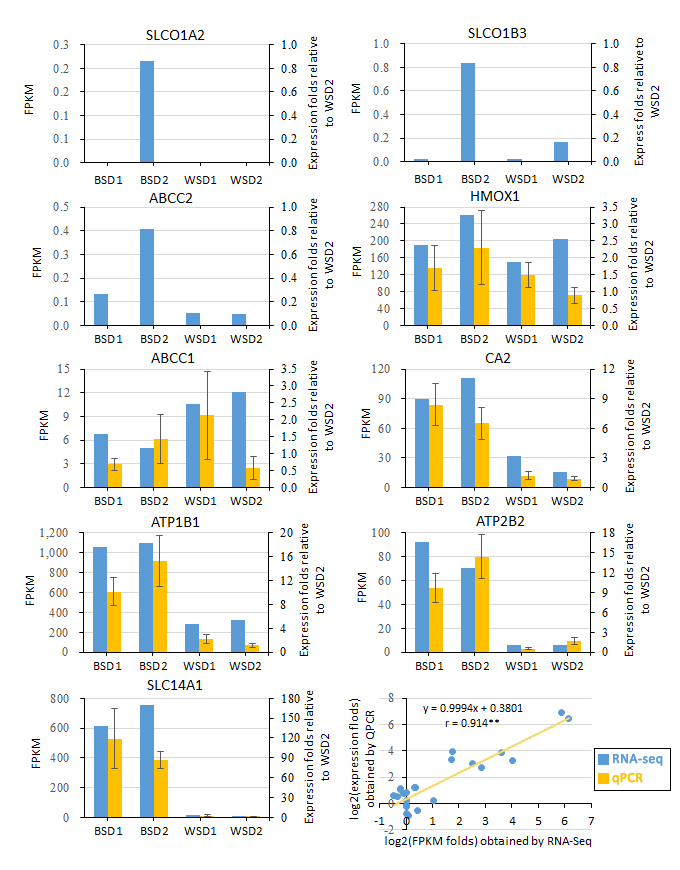

Supplement: Supplementary file 6 — Verification of RNA-seq results using qPCR. BSD1 & BSD2 respectively indicate two blue-shelled duck groups; accordingly WSD1 & WSD2 represent two white-shelled duck groups. In the RNA-seq, FPKM values in each group were calculated by Cuffdiff using three biological replicates. In the qPCR, the WDS2 group was set as the criterion; the abundances of genes in each group were presented as expression folds relative to the criterion. The qPCR results were expressed as mean ± SD obtained from 3 biological replicates. The correlation plot showed the relationship between qPCR and RNA-seq results of HMOX1, ABCC1, CA2, ATP1B1, ATP2B2 and SLC14A1. ** indicates a significant correlation relationship at P < 0.01. (JPEG 367 kb) [file 12864_2017_4135_MOESM6_ESM.jpg]
